# Serum Uric Acid Levels Are Associated with the Echogenic Features of Carotid Plaque Vulnerability in Elderly Patients with Atherosclerotic Disease

**DOI:** 10.3390/metabo13060693

**Published:** 2023-05-26

**Authors:** Daniela Mastroiacovo, Evaristo Ettorre, Alessandro Mengozzi, Agostino Virdis, Antonio Camerota, Mario Muselli, Stefano Necozione, Raffaella Bocale, Claudio Ferri, Giovambattista Desideri

**Affiliations:** 1 Angiology Unit, Medical Department, “SS. Filippo and Nicola” Hospital, 67051 L’Aquila, Italy; daniela.mastroiacovo@gmail.com; 2 Department of Clinical, Internal Medicine, Anesthesiologic and Cardiovascular Sciences, Sapienza University of Rome, 00161 Rome, Italy; evaristo.ettorre@uniroma1.it; 3 Department of Clinical and Experimental Medicine, University of Pisa, 56126 Pisa, Italy; alessandro.mengozzi@medmcs.unipi.it (A.M.); agostino.virdis@unipi.it (A.V.); 4 Center for Translational and Experimental Cardiology (CTEC), Department of Cardiology, Zurich University Hospital, University of Zurich, 8952 Schlieren, Switzerland; 5 Institute of Life Sciences, Scuola Superiore Sant’Anna, 56126 Pisa, Italy; 6 Department of Life, Health and Environmental Sciences, University of L’Aquila, 67100 L’Aquila, Italy; a.camerota@ausl.latina.it (A.C.); mario.muselli@univaq.it (M.M.); stefano.necozione@univaq.it (S.N.); claudio.ferri@univaq.it (C.F.); 7 Division of Endocrine Surgery, Agostino Gemelli University Hospital Foundation Scientific Institute for Research, Hospitalization and Healthcare (IRCCS), Catholic University of the Sacred Heart, 00168 Rome, Italy; raffaella.bocale@policlinicogemelli.it

**Keywords:** serum uric acid, carotid atherosclerotic plaque, grayscale median

## Abstract

Uric acid is a marker of inflammation and a risk factor for atherosclerosis that has been suggested to play a role in carotid plaque instability. Reduced atherosclerotic plaque echogenicity at ultrasound examination is associated with alarming histopathological features and inflammation. In this study, we investigated the relationship between serum uric acid (SUA) levels and echogenic patterns of plaque instability in elderly subjects with carotid atherosclerosis. Since uric acid metabolism largely depends on renal function, SUA levels were indexed for serum creatinine levels (SUA/SCr). We enrolled 108 patients aged 65 years or more (72.7 ± 5.9 years; 50 females and 58 males) who underwent carotid duplex ultrasound to evaluate plaque echogenicity by greyscale median (GSM). The regression analysis demonstrated a significant inverse association between the GSM and the SUA/SCr ratio (β: −0.567; 95% CI −0.751 to −0.384 and *p* < 0.0001). Stepwise multivariate regression showed that the SUA/SCr ratio explained 30.3% of GSM variability (β: −0.600; 95% CI −0.777/−0.424, *p* < 0.0001, and semi-partial correlation 0.303). After a mean period of 3.5 ± 0.5 years, 48 patients were reevaluated according to the same baseline study protocol. The regression analysis demonstrated a still significant inverse association between the GSM and the SUA/SCr ratio (β: −0.462; 95% CI −0.745 to −0.178 and *p* = 0.002). Stepwise multivariate regression showed that the SUA/SCr ratio explained 28.0% of GSM variability (coefficient −0.584, 95% CI −0.848/−0.319, *p* < 0.0001, and semi-partial R^2^ 0.280). In conclusion, this study demonstrates that SUA levels indexed for serum creatinine are associated with the echogenic features of carotid plaque vulnerability in elderly patients with atherosclerotic disease. These data could suggest an influential role for uric acid metabolism in carotid plaque biology.

## 1. Introduction

Uric acid is the final product of purine metabolism and is synthesized by the enzyme xanthine oxidase through the oxidation of xanthine and hypoxanthine. The association between serum uric acid (SUA) and atherosclerotic cardiovascular disease has been investigated for almost 50 years [1]. A growing body of evidence suggests that uric acid metabolism could be involved in the pathophysiology of atherosclerosis by promoting endothelial activation/dysfunction and proatherogenic inflammation [2]. Uric acid expression in carotid plaques has been recently demonstrated to be positively correlated with SUA levels and associated with inflammatory markers expressed in carotid plaques [3]. In particular, patients with vulnerable carotid plaques leading to an ipsilateral symptomatic cerebrovascular event have a higher concentration of uric acid in the carotid plaque and higher SUA compared with asymptomatic patients [3]. These findings support a pathophysiological role for uric acid in the pathogenesis of carotid atherosclerosis and possibly an influential role as a promoter of atherosclerotic plaque vulnerability. Some recent papers have proposed the indexing of SUA to renal function, as measured by serum creatine, to improve its prognostic role for cardiovascular disease, mainly in some categories of people such as patients with diabetes, nephropathy, or chronic pulmonary disease or in menopausal women [4,5,6,7,8,9]. As a matter of fact, due to the pivotal role of the kidney in modulating uric acid excretion, the indexing of SUA for renal function could shift the same SUA level toward a more dangerous pathophysiological pattern [10].

Currently, besides the degree of carotid stenosis, the focus of research on atherosclerotic plaque imaging is on the functional state of the lesion [11]. Indeed, the recognition of vulnerable carotid plaques, presenting one or more characteristics such as plaque echolucency, intra-plaque hemorrhage, stenosis progression, large area, large juxta-luminal black area, impaired cerebral vascular reserve, and spontaneous embolization is a means of identifying apparently healthy patients at risk of future ischemic events [12,13,14]. Vulnerable plaque formation is recognized to be a multifactorial process. Inflammation, lipid accumulation, apoptosis, proteolysis, thrombotic processes, and angiogenesis are among the main determinants of carotid plaque vulnerability [15]. Several different ultrasound techniques can reliably assess the features of the vulnerability of carotid plaques [16]. Among these, the grayscale median (GSM) analysis represents a standardized method that, through digital image examination, allows for a quantitative and objective assessment of the echolucency of the plaque, considering the overall median value of the atherosclerotic plaque [16,17]. Indeed, using GSM analyses, several studies have demonstrated a correlation between low echogenicity and alarming histopathological plaque features [18,19,20] or an association between plaque echolucency and inflammation [21].

In this study, we investigated the association between the echogenic features of carotid atherosclerotic plaques, as assessed by GSM, and SUA levels indexed for renal function in patients with carotid atherosclerotic plaques.

## 2. Materials and Methods

### 2.1. Subjects

We analyzed data from elderly subjects (aged 65 years or more) with carotid atherosclerosis enrolled in a previous study conducted by our group aiming to evaluate the relationship between echogenicity of carotid plaques, as assessed by GSM analysis, and cognitive performance in elderly patients with no history of cerebrovascular events and/or clinical evidence of dementia [22]. Briefly, participants were recruited among those consecutively referred to our Angiology Unit with a history of known or suspected carotid artery atherosclerotic disease, after ultrasonographic demonstration of atherosclerotic plaques, defined as at least a focal thickness >1.5 mm as measured from the intima–lumen to the media–adventitia interfaces [23]. The exclusion criteria were age <65 years, history and symptoms or signs of neurological disease and cerebrovascular events (transient ischemic attack or stroke), or neuroradiological evidence, if available, of vascular brain lesions, previous carotid endarterectomy, previous neck irradiation, presence of major depressive states, psychiatric disorders, and overt cognitive dysfunction. All participants underwent a complete clinical evaluation, including a comprehensive medical history and blood samplings after an overnight fasting period for routine serum biochemistry tests, including lipid profile and fasting plasma glucose. Clinical systolic and diastolic blood pressures were assessed in the morning with the use of a validated oscillometric device with appropriately sized cuffs (Omron HEM 7155-E; Omron Matsusaka Co., Ltd., Kyoto, Japan) on the non-dominant upper arm. Blood pressure was measured after the participant had rested for 15 min in a seated position. The first blood pressure measurement was taken but discarded, and the subsequent three consecutive blood pressure readings, taken at 3-min intervals, were recorded. The average of these latter measurements was considered for statistical analysis.

All participants underwent ultrasound evaluation of neck vessels to assess the degree of stenosis and plaque echogenicity. Starting from the third year after the first evaluation, participants were re-evaluated according to the same procedures used at baseline. In the present report, we analyzed data from 108 subjects for whom serum uric acid levels were available.

### 2.2. Laboratory Analysis

Clinical chemistry evaluations, including uric acid, creatinine, and lipid profile, were performed using an Alinity CI-series system (Abbott Laboratories, Chicago, IL, USA). The estimated glomerular filtration rate (eGFR) was calculated according to the CKD-EPI (Chronic Kidney Disease Epidemiology Collaboration) equation [24].

### 2.3. Ultrasound Method

The ultrasound examinations were performed by an experienced sonographer using a Siemens Acuson Sequoia 512 ultrasound machine (Siemens Healthcare s.r.l., Milano, Italy) and a 7–10 MHz high-frequency linear probe. All patients were examined supine with a slight head tilt. Anterior, lateral, and posterior projections were used to image the plaque longitudinally. The largest plaque visualized with an optimal projection at the carotid bifurcation, or the proximal internal carotid artery, was chosen for the assessment of plaque echogenicity. In the case of hypoechoic or anechoic plaques, one image with a color Doppler or power Doppler was saved to ensure the correct delineation of the plaque margin. The settings for the ultrasound machine were adjusted and standardized for all examinations by using a maximum dynamic range (60 dB) and by setting the gain to ensure an almost noiseless vessel lumen (blood) and an echo-attenuated area of adventitia. B-mode and corresponding color Doppler images were saved in digital form on a magneto-optical disk. All appropriate images were stored in and processed by a computer. Additionally, the degree of stenosis based on B-mode images was measured according to the North American Symptomatic Carotid Endarterectomy Trial (NASCET) method [25].

### 2.4. Plaque Echogenicity

The GSM analysis was performed by the same operator, who was blinded to the clinical profile of the patients. Images were analyzed using a graphics program (Adobe Photoshop 5.0, Adobe Systems Incorporated) with a 2- to 3-fold increase in initial size. According to a previously described and validated methodology [19,26], the color information in the digitized image was omitted so that all processing and analysis were performed on images in gray mode. The linear scale of the “curves” option of the software was adjusted to achieve values of the blood between 0 and 5 and gray values of the adventitia between 185 and 195. In these normalized images, each plaque was outlined using the computer mouse, and its grayscale content was analyzed for the mean, standard deviation, median, and total pixel calculation, using the histogram feature. The GSM, which represents the median of the frequency distribution of tones of pixels included in the plaque areas, was used as a measure of the overall plaque echogenicity.

### 2.5. Statistical Analysis

The normality of the distributions of the variables was evaluated with the Shapiro–Wilk test. Paired comparisons between continuous variables were performed using the Wilcoxon signed-rank test. Spearman’s rank correlation was used to assess relationships between variables. Univariate linear regression analysis was conducted to describe the relationship between the GSM, the dependent variable, and the SUA/SCr ratio. Multivariate linear regression analysis was conducted with the stepwise method to identify independent variables correlated with GSM. For both univariate and multivariate models of linear regression, the assumption of normality of the distribution of the residuals (the differences between the observations and the estimated values) was evaluated with the Shapiro–Wilk test. Continuous variables are presented as mean ± standard deviation, while categorical variables are presented as percentages. A *p*-value < 0.05 was considered statistically significant when comparing variables. All the statistical analyses were performed using SAS version 9.4 (SAS Institute Inc., Cary, NC, USA) and the Medcalc^®^ Statistical Software version 20.126 (MedCalc Software Ltd., Ostend, Belgium). G*Power 3.1.9.6 software was used to perform post hoc power analysis. A sample size of 108 subjects had a statistical power greater than 90% in identifying a significant statistical correlation coefficient (rho) = 0 −0.497 (error = 0.05, two-tailed test) in the relationship between the GSM and SUA/Scr. A test of equality for correlation coefficients was used to test differences between them. Unless otherwise specified, the data are presented as mean ± standard deviation.

## 3. Results

A total of 108 elderly patients (72.7 ± 5.9 years; 50 females and 58 males) with carotid atherosclerotic disease were evaluated. The general characteristics of the study population are shown in Table 1. Hypertension and hypercholesterolemia were the most prevalent cardiovascular risk factors (81.5% and 73.2%, respectively). Among the drugs potentially influencing the study outcome [13,27] were statins (43.9%), angiotensin-converting enzyme inhibitors (38.8%), angiotensin receptor blockers (29.6%), beta-blockers (25.5%), calcium channel blockers (23.5%), diuretics (45.9%), and antiplatelet agents (58.3%).

The GSM values were inversely correlated with both SUA (rho: −0.439; *p* < 0.0001) and the SUA/SCr ratio values (rho: −0.497; *p* < 0.0001) (Table 2). Although the difference between these two correlations was not statistically significant (*p* = 0.591), due to the high collinearity between SUA and the SUA/SCr ratio (rho: 0.825; *p* < 0.0001), SUA values were not considered in subsequent analyses aiming to evaluate the independent predictors of the GSM. An inverse correlation between the GSM and the NASCET score was also found (rho: −0.187; *p* = 0.05).

**Figure 1:**
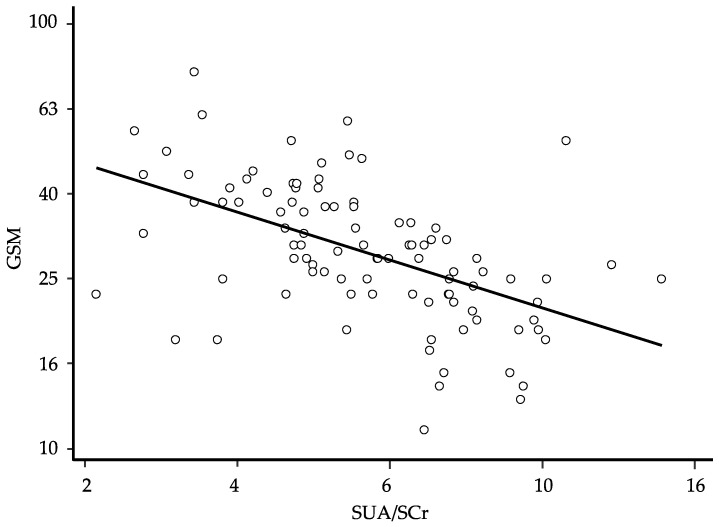
Relationship between the grayscale median (GSM) and the serum uric acid/serum creatinine (SUA/SCr) ratio at baseline; regression line fitted after logarithmic transformation of the data and elimination of 10 residual outliers to ensure the normal distribution of the residues (β: −0.567; 95% CI −0.751 to −0.384, *p* < 0.0001, and *n* = 98); for ease of interpretation, the results of calculations are back-transformed to their original scale.

In order to define the degree of prediction of the GSM by the SUA/SCr ratio, we performed a univariate linear regression. After logarithmic transformation of the data and elimination of the residual outliers (*n* = 10) to ensure the normal distribution of the residues, the univariate regression showed a significant association between the GSM score and the SUA/SCr ratio value (β: −0.567; 95% CI −0.751 to −0.384 and *p* < 0.0001) (Figure 1). The general characteristics of the study participants after the exclusion of the 10 residual outliers are presented in Table 3.

With the aim of identifying the possible predictors of the GSM, we conducted a stepwise multivariate linear regression. The model showed that female gender, higher SUA/SCr ratio value, and higher NASCET score were associated with a lower GSM score (Table 4). The semi-partial correlation showed that the SUA/SCr ratio was the variable most strongly associated with the GSM, explaining 30.3% of GSM variability. This association slightly improved, although not significantly, by adding eGFR to the model (coefficient −0.738, 95% CI −0.933 to −0.544, *p* < 0.0001, and semi-partial R^2^ 0.351). None of the pharmacological treatments considered was significantly associated with the GSM values.

At follow-up, of the 98 patients analyzed in the cross-sectional phase of the study, 29 subjects had undergone carotid endarterectomy and were excluded. Of the 69 patients kept on medical treatment, 6 had died (5 cardiovascular deaths), 8 had experienced cerebrovascular or cardiovascular events, and 7 declined to attend the follow-up evaluation. Thus, a longitudinal analysis was performed on the remaining 48 patients (29 females and 19 males) after a mean period of 3.5 ± 0.5 years. No significant variations in the GSM and SUA/SCr values were observed in the subgroup of patients re-evaluated at follow-up (Table 5). On the contrary, both the NASCET values and eGFR were slightly but significantly worse at follow-up. The GSM values were still inversely correlated with both SUA levels (rho: −0.361; *p* = 0.008) and the SUA/SCr ratio values (rho: −0.375; *p* = 0.006) (Table 6). The difference between these two correlations was not statistically significant (*p* = 0.939).

**Figure 2:**
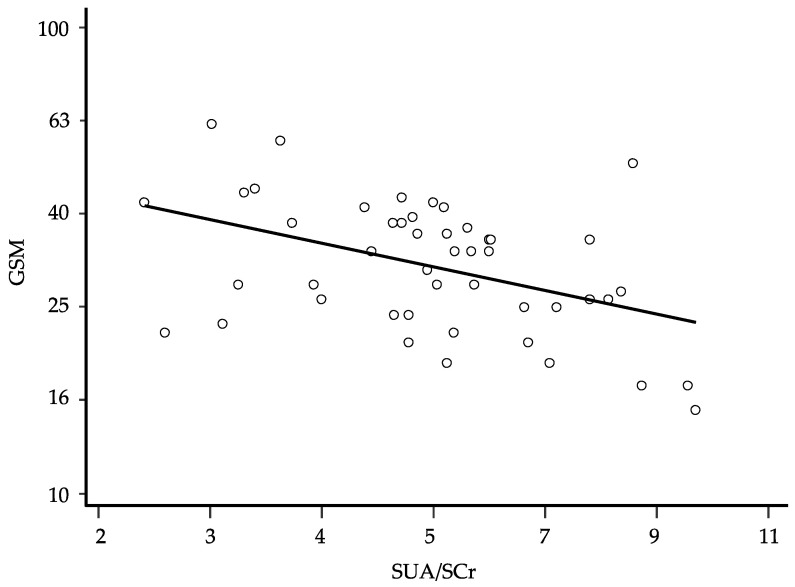
Relationship between the grayscale median (GSM) and the serum uric acid/serum creatinine (SUA/SCr) ratio at follow-up; regression line fitted after logarithmic transformation of the data (β: −0.462; *p* = 0.002, 95% CI −0.745 to −0.178, and *n* = 48); for ease of interpretation, the results of the calculations are back-transformed to their original scale.

After logarithmic transformation of the data, univariate linear regression showed that also in this latter analysis, the GSM was inversely associated with the SUA/SCr ratio (β: −0.462; 95% CI −0.745 to −0.178 and *p* = 0.002) (Figure 2). The stepwise multivariate regression showed again that female gender, higher SUA/SCr ratio, and higher NASCET were associated with a lower GSM score (Table 7). The semi-partial correlation showed that the SUA/SCr ratio was the variable most strongly associated with the GSM, explaining 28.0% of GSM variability. Even at this time, this association improved slightly, although not significantly, by adding eGFR to the model (coefficient −0.683, 95% CI −1.016 to −0.350, *p* = 0.0002, and semi-partial R^2^ 0.242). According to the logistic regression model, the basal SUA/SCr values did not predict the need for endarterectomy or other considered events (death and cerebrovascular or cardiovascular events). None of the treatments considered was significantly associated with the GSM values.

## 4. Discussion

The present report demonstrates a significant relationship between predominantly echolucent plaques and SUA levels indexed for renal function in elderly patients with carotid atherosclerosis. To the best of our knowledge, this study is the first one to demonstrate such intriguing associations.

The potential involvement of uric acid metabolism in the pathophysiological mechanisms underlying atherosclerotic plaque development and progression up to its complications has been suggested by several lines of evidence [28,29,30,31,32,33,34]. From a pathophysiological perspective, uric acid seems to have all the biological potential to influence atherogenesis because of its ability to promote endothelial dysfunction, vascular damage, platelet adhesiveness, and activation of the renin–angiotensin system as well as the activation of proliferative and inflammatory pathways in the vascular smooth muscle cells [2]. Furthermore, uric acid expression in carotid plaques has been demonstrated to be positively correlated with SUA levels and associated with inflammatory markers expressed in carotid plaques [3]. The evidence that urate-lowering treatment with xanthine oxidase inhibitors is able to blunt the progression of arterial stiffness in patients with gout, elevated SUA levels, and moderate to high cardiovascular risk profile [35] further supports the hypothesis that uric acid may determine plaque vulnerability and subsequent rupture. The evidence of a direct relationship between SUA levels and carotid plaque echolucency that we have found in elderly subjects with atherosclerotic disease provides further, indirect, support to the hypothesis of a possible influential role of this product of purine metabolism in plaque biology. Indeed, plaque echolucency is the imaging correlated to histopathologic evidence of either lipid-rich necrotic core and/or intraplaque hemorrhage [36,37].

It is noteworthy that our paper particularly takes into consideration the indexing of SUA to renal function represented by serum creatinine. Because of the pivotal role of the kidney in modulating uric acid excretion, the normalization of SUA for serum creatinine allows for the minimization of the influence of different degrees of renal dysfunction on SUA levels. This aspect appears particularly interesting in light of the growing body of evidence suggesting that the association between uric acid metabolism and atherosclerotic cardiovascular disease could be deeply influenced not only by the uric acid molecule but also by the metabolic pathway generating uric acid [10]. As a matter of fact, xanthine oxidase—the key enzyme that catalyzes the oxidation of hypoxanthine to xanthine, and further catalyzes xanthine to uric acid—has all the biological capability to deeply influence the development of cardiovascular and metabolic diseases [2,10,38]. Worth mentioning in this regard is the recent demonstration by Ganji et al. [39] that carotid atherosclerotic plaques from symptomatic patients were associated with significantly higher xanthine oxidase expression in macrophages versus asymptomatic plaques. Even more interesting, the percentage of local expression of xanthine oxidase in atherosclerotic plaques was positively correlated to SUA levels, which were similar to those observed in our report. Thus, the indexing of SUA for renal function could shift the same SUA level toward a more dangerous pathophysiological pattern. Although this hypothesis could appear too speculative, several recent papers have proposed the SUA/SCr ratio as a new variable able to explore cardiovascular risk, mainly in some categories of people such as patients with diabetes, nephropathy, or chronic pulmonary disease, or menopausal women [4,5,6,7,8,9]. It is interesting to note that the significant relationship between SUA levels and carotid plaque echolucency that we have found in our study was tendentially increased after SUA indexing for renal function, although the difference between the two correlations was not statistically different. Even more interesting, when eGFR as a further marker of renal function was added to the model, the coefficient of association between the GSM and SUA/SCr increased slightly, although not significantly. The significant predictive role of female gender for the GSM value that we have found in our study could further support this data interpretation. Indeed, a stronger predictive effect of serum UA on cardiovascular events has been demonstrated in female patients, suggesting a predominance of the uric-acid-generating pathway in women beyond the relevant uricosuric effects of estrogens [30]. The persistence of the association between the GSM and SUA at the end of the follow-up provides further evidence of the robustness of our study findings. After a mean of 3.5 years, SUA levels indexed for serum creatinine still explained 28% of GSM variability despite the consistent reduction in the study population. Although our study did not investigate the pathophysiological mechanism underlying the association between SUA levels indexed for renal function and GSM values, our finding might suggest a predominant pathophysiological role for the uric-acid-generating pathway.

This study has some limitations. First, we have to consider that the GSM is a median value of pixel brightness of the entire carotid plaque, not taking into consideration focal variability and different patterns of echogenicity [26]. Second, the GSM approach used to assess plaque echogenicity may be biased by the subjectivity in outlining the plaques before GSM analysis is performed, although studies have shown a reasonable intra- and interobserver variability [40,41]. Third, we have to underline that the follow-up analysis was performed in about half of the initial cohort. Thus, we cannot exclude a potential bias deriving from the low number of subjects re-analyzed at follow-up. Finally, association does not prove causation. Thus, our data could suggest, but not prove, an influential role for uric acid in atherosclerotic plaque biology. On the other hand, our study was designed to start from a pathophysiological perspective rather than with the purpose of identifying a prognostic marker.

## 5. Conclusions

The present study provides the first evidence that serum uric acid levels are associated with the echogenic features of carotid plaque vulnerability in elderly patients with atherosclerotic disease. These data could suggest an influential role for uric acid metabolism in carotid plaque biology.

## Figures and Tables

**Table 1:** General characteristics of the study population at baseline (*n* = 108).

Age	72.7 ± 5.9
Gender (males/females)	58/50
BMI (kg/m^2^)	27.5 ± 4.1
Total cholesterol (mg/L)	196.9 ± 46.6
HDL-C (mg/dL)	47.9 ± 12.5
LDL-C (mg/dL)	118.3 ± 44.1
Triglyceride (mg/dL)	156.0 ± 69.2
SBP (mmHg)	139.2 ± 14.7
DBP (mmHg)	78.5 ± 7.6
GSM	31.5 ± 13.7
NASCET (%)	44.4 ± 20.9
Hypertension, *n* (%)	88 (81.5%)
Hypercholesterolemia, *n* (%)	79 (73.2%)
Diabetes mellitus, *n* (%)	22 (20.4%)
Smoking, *n* (%)	16 (14.8%)
Creatinine (mg/dL)	1.0 ± 0.2
eGFR (mL/min/1.73 m^2^)	71.1 ± 16.7
SUA (mg/dL)	6.1 ± 1.8
SUA/SCr ratio	6.2 ± 2.1

BMI: body mass index; HDL-C: high-density lipoprotein cholesterol; LDL-C: low-density lipoprotein cholesterol; SBP: systolic blood pressure; DBP: diastolic blood pressure; GSM: grayscale median; NASCET: North American Symptomatic Carotid Endarterectomy Trial; eGFR: estimated glomerular filtration rate; SUA: serum uric acid; SCr: serum creatinine.

**Table 2:** Spearman’s correlation at baseline between GSM and other analyzed variables (*n* = 108).

	Coefficient	*p*-Value
Age	−0.076	0.434
BMI	0.053	0.585
Total cholesterol	−0.082	0.399
HDL-C	0.060	0.541
LDL-C	−0.037	0.706
Triglyceride	−0.109	0.262
SBP	−0.072	0.459
DBP	0.028	0.776
NASCET	−0.187	0.050
Creatinine	0.147	0.129
eGFR	−0.075	0.441
SUA	−0.439	<0.0001
SUA/SCr ratio	−0.497	<0.0001

GSM: grayscale median; BMI: body mass index; HDL-C: high-density lipoprotein cholesterol; LDL-C: low-density lipoprotein cholesterol; SBP: systolic blood pressure; DBP: diastolic blood pressure; NASCET: North American Symptomatic Carotid Endarterectomy Trial; eGFR: estimated glomerular filtration rate; SUA/SCr: serum uric acid/serum creatinine.

**Table 3:** General characteristics of the study population after the exclusion of ten residual outliers (*n* = 98).

Age	72.7 ± 5.9
Gender (males/females)	53/45
BMI (kg/m^2^)	27.6 ± 4.0
Total cholesterol (mg/L)	197.5 ± 47.6
HDL-C (mg/dL)	47.8 ± 12.1
LDL-C (mg/dL)	119.1 ± 44.9
Triglyceride (mg/dL)	155.2 ± 70.2
SBP (mmHg)	139.5 ± 15.1
DBP (mmHg)	78.6 ± 7.4
GSM	30.9 ± 11.8
NASCET (%)	44.3 ± 20.9
Hypertension, *n* (%)	81 (82.7%)
Hypercholesterolemia, *n* (%)	70 (71.4%)
Diabetes mellitus, *n* (%)	19 (19.4%)
Smoking, *n* (%)	11 (11.2%)
Creatinine (mg/dL)	1.0 ± 0.2
eGFR (mL/min/1.73 m^2^)	72.1 ± 16.6
SUA (mg/dL)	6.0 ± 1.7
SUA/SCr ratio	6.2 ± 2.2

BMI: body mass index; HDL: high-density lipoprotein cholesterol; LDL: low-density lipoprotein cholesterol; SBP: systolic blood pressure; DBP: diastolic blood pressure; GSM: grayscale median; NASCET: North American Symptomatic Carotid Endarterectomy Trial; eGFR: estimated glomerular filtration rate; SUA: serum uric acid; SCr: serum creatinine.

**Table 4:** Multiple linear regression analysis for the prediction of the GSM at baseline.

	Coefficient	95% CI	*p*-Value	Semi-Partial R^2^
Gender	−0.068	−0.122; −0.143	0.014	0.042
SUA/SCr ratio	−0.600	−0.777; −0.424	<0.0001	0.303
NASCET	−0.208	−0.335; −0.082	0.002	0.071

GSM: grayscale median; SUA: serum uric acid; SCr: serum creatinine; NASCET: North American Symptomatic Carotid Endarterectomy Trial. Multiple linear regression with stepwise selection of independent variables after logarithmic transformations of the data.

**Table 5:** General characteristics of the study participants who were re-evaluated at the end of follow-up (*n* = 48).

	Baseline	Follow-Up	*p*
Age	71.0 ± 5.6	74.2 ± 5.8	<0.0001
BMI (kg/m^2^)	27.7 ± 4.1	27.8 ± 4.2	0.689
Total cholesterol (mg/dL)	209.1 ± 49.9	198.5 ± 42.	0.068
HDL-C (mg/dL)	50.6 ± 13.0	48.6 ± 12.0	0.089
LDL-C (mg/dL)	129.2 ± 500	120.9 ± 37.5	0.104
Triglyceride (mg/dL)	151.8 ± 62.9	149.5 ± 53.7	0.886
SBP (mmHg)	138.6 ± 15.4	136.4 ± 14.6	0.485
DBP (mmHg)	79.5 ± 8.1	77.2 ± 7.1	0.040
GSM	32.4 ± 10.5	32.1 ± 10.3	0.599
NASCET score	32.7 ± 12.6	37.6 ± 13.5	<0.0001
Creatinine (mg/dL)	1.0 ± 0.2	1.0 ± 0.2	0.266
eGFR (ml/min/1.73 m^2^)	69.4 ± 17.3	67.2 ± 16.0	0.018
SUA (mg/dL)	5.6 ± 1.5	5.5 ± 1.2	0.770
SUA/SCr ratio	5.8 ± 1.9	5.6 ± 1.7	0.637

BMI: body mass index; HDL: high-density lipoprotein cholesterol; LDL: low-density lipoprotein cholesterol; SBP: systolic blood pressure; DBP: diastolic blood pressure; GSM: grayscale median; NASCET: North American Symptomatic Carotid Endarterectomy Trial; eGFR: estimated glomerular filtration rate; SUA: serum uric acid; SCr: serum creatinine.

**Table 6:** Spearman’s correlation at follow-up between the GSM and other analyzed variables.

	Coefficient	*p*-Value
Age	−0.117	0.404
BMI	0.160	0.253
Total cholesterol	0.007	0.963
HDL-C	−0.010	0.943
LDL-C	0.018	0.901
Triglyceride	−0.072	0.610
SBP	−0.056	0.693
DBP	0.129	0.357
NASCET	−0.170	0.248
Creatinine	0.143	0.308
eGFR	0.065	0.645
SUA (mg/dL)	−0.361	0.008
SUA/SCr ratio	−0.375	0.006

GSM: grayscale median; BMI: body mass index; HDL-C: high-density lipoprotein cholesterol; LDL-C: low-density lipoprotein cholesterol; SBP: systolic blood pressure; DBP: diastolic blood pressure; NASCET: North American Symptomatic Carotid Endarterectomy Trial; eGFR: estimated glomerular filtration rate; SUA: serum uric acid; SCr: serum creatinine.

**Table 7:** Multiple linear regression analysis for the prediction of the GSM at follow-up.

	Coefficient	95% CI	*p*-Value	Semi-Partial R^2^
Gender (female)	−0.087	−0.155; −0.018	0.014	0.092
SUA/SCr ratio	−0.584	−0.848; −0.319	<0.0001	0.280
NASCET	−0.273	−0.489; −0.058	0.014	0.093

GSM: grayscale median; SUA: serum uric acid; SCr: serum creatinine; NASCET: North American Symptomatic Carotid Endarterectomy Trial. Multiple linear regression with stepwise selection of independent variables after logarithmic transformation of the data.

## Data Availability

The data are available from the investigators upon reasonable request. The data are not publicly available due to specific restrictions.
